# Demographic stochasticity drives epidemiological patterns in wildlife with implications for diseases and population management

**DOI:** 10.1038/s41598-018-34623-0

**Published:** 2018-11-15

**Authors:** Sébastien Lambert, Pauline Ezanno, Mathieu Garel, Emmanuelle Gilot-Fromont

**Affiliations:** 1 Université de Lyon, Université Lyon 1, UMR CNRS 5558 Laboratoire de Biométrie et Biologie Evolutive, Villeurbanne, France; 2 Office National de la Chasse et de la Faune Sauvage, Unité Ongulés Sauvages, 5 allée de Bethléem – ZI Mayencin, 38610 Gières, France; 3 BIOEPAR, INRA, Oniris, 44307 Nantes, France; 4 Université de Lyon, VetAgro Sup, Marcy l’Etoile, France

## Abstract

Infectious diseases raise many concerns for wildlife and new insights must be gained to manage infected populations. Wild ungulates provide opportunities to gain such insights as they host many pathogens. Using modelling and data collected from an intensively monitored population of Pyrenean chamois, we investigated the role of stochastic processes in governing epidemiological patterns of pestivirus spread in both protected and hunted populations. We showed that demographic stochasticity led to three epidemiological outcomes: early infection fade-out, epidemic outbreaks with population collapse, either followed by virus extinction or by endemic situations. Without re-introduction, the virus faded out in >50% of replications within 4 years and did not persist >20 years. Test-and-cull of infected animals and vaccination had limited effects relative to the efforts devoted, especially in hunted populations in which only quota reduction somewhat improve population recovery. Success of these strategies also relied on the maintenance of a high level of surveillance of hunter-harvested animals. Our findings suggested that, while surveillance and maintenance of population levels at intermediate densities to avoid large epidemics are useful at any time, a ‘do nothing’ approach during epidemics could be the ‘least bad’ management strategy in populations of ungulates species facing pestivirus infection.

## Introduction

The emergence and persistence of infectious diseases in wildlife are of increasing concern^1,2^, as they represent a threat to public health (*e.g*., rabies, avian influenza), cause economic and food safety issues in veterinary health (*e.g*., bovine tuberculosis) and represent conservation issues (*e.g*., facial tumour disease in Tasmanian devils *Sarcophilus harrisii*)^3^. The key role of wildlife in disease emergence contrasts with the paucity of management options of known efficacy when epidemics emerge in wildlife^4^ and brings to light the need to better understand pathogen invasion and persistence in wildlife populations and to identify relevant options for disease management. When management options are carried out in the field, they should be monitored in an active adaptive management approach in order to improve scientific knowledge during the management process^5^. For example, the recent assessment concerning brucellosis in the Greater Yellowstone Area underlined that several management actions were not monitored for scientific assessment of effectiveness, which led to uncertainty on the efficacy of some measures and slower learning process^5^.

Unlike domestic populations, wildlife interacts with a varied and unpredictable environment^6^, and has complex processes of population dynamics that may affect disease emergence and propagation^7^. Identifying the factors that drive pathogen invasion and persistence in wildlife thus requires better accounting for the biological and ecological characteristics of the host populations^7–9^.To this end, mathematical modelling is often the only way to compare management strategies in such populations, as experimental approaches can rarely be implemented. Modelling has been used successfully in the past, in a non-epidemiological context, to predict optimal harvesting strategies of exploited populations^10–12^. In particular, given the importance of environmental variability on population dynamics, recent modelling approaches have shown that the demography of structured populations is better taken into account by stochastic than deterministic approaches^13^. Beyond environmental stochasticity, demographic stochasticity, defined as the variation in dynamics of small populations owing to the probabilistic nature of individual processes, such as birth, death or pathogen transmission^14^, can also be in play in small populations. In particular, stochastic processes generate a risk of population/virus extinction^15,16^ that also needs to be accounted for when predicting epidemiological outcomes and assessing related management issues. Predicting the emergence and persistence of pathogens in wildlife populations and the related efficacy of their management thus requires that the complex interplay between contact structure, pathogen virulence and the unpredictable inter-annual variation in population growth rates be taken into account^9,17^.

Stochastic epidemiological modelling can help investigate such complex wildlife disease system and understand the underlying processes of pathogen transmission^8,18^. It provides an integrated mechanistic representation of the system, proven to be useful in testing for biological assumptions^19^ and assessing disease management strategies^20,21^. For instance, evaluation with stochastic models has been used to demonstrate that populations could be protected effectively at lower cost by targeting only large outbreaks^22^, or to reassess current strategies when they appear ineffective^23^.

Among wild-living species, large herbivores are keystone species that shape the structure, diversity, and functioning of most terrestrial ecosystems^24^ and provide substantial resources, supplying rural communities with goods and economic income^25^. The management of large herbivore species relies on a sound knowledge of population biology, that should include the effects of management on diseases in natural populations^26^, and particularly emerging diseases, which are a potential threat to these animals^27^. Among emerging wildlife diseases threatening large herbivores, pestiviruses are relevant biological models of epizootics caused by domestic-wildlife transmission as documented in numerous species^28^, in particular in wild boar (*Sus scrofa*)^29^ and Pyrenean chamois (*Rupicapra pyrenaica pyrenaica*)^30^. The latter species is an emblematic ungulate in the Pyrenean mountain, distributed widely from west to east of the chain. In 2010–2011, the estimated minimum population size was around 31,160 in France, 22,799 in Spain and 666 in Andorra^31^. While some populations are not hunted such as in the Pyrenean National Park in France^32^, most are under game use with hunting rates varying between 5–15%. Populations are managed mainly by hunting federations in France, and by National Game Reserves in Spain^33^. In this species, the first outbreak of pestivirus, reported in 2001 in Spain^34^, caused a 42% decrease in population size^30^. The virus was typed as Border Disease Virus (BDV) of genotype BDV-4^35–37^. New cases then occurred in Spain, the Principality of Andorra and France, leading to multiple outbreaks that caused major decreases in Pyrenean chamois populations^38–40^, while the infection was expanding westward^41,42^. However, later investigations traced back the entry of pestivirus in chamois populations between 1989 and 1991^42–44^.

**Figure 1:**
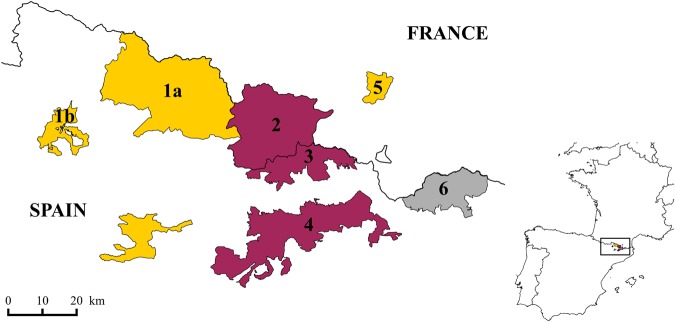
Map of Eastern Pyrenees showing for Pyrenean chamois the most studied hunting reserves in France and Spain (QGIS Development Team (2018). QGIS Geographic Information System. Open Source Geospatial Foundation Project. http://qgis.osgeo.org^99^). 1a: Alt Pallars-Aran National Hunting Reserve (NHR), Northern Sector; 1b: Alt Pallars-Aran NHR, Southern Sector (Boí); 2: Principality of Andorra; 3: Cerdanya-Alt Urgell NHR; 4: Cadí NHR; 5: National Game and Wildlife Reserve of Orlu; 6: Freser-Setcases NHR. In purple (cluster 2): severe outbreaks followed by quick population recovery and decrease in virus circulation^38,39^. In yellow (cluster 3): outbreaks followed by decreasing trends or absence of population recovery and an endemic situation of the infection^30,40,41^. In grey: persistence of the virus without any negative impact on population size^39,41^. See text and Figure 2 for the definition of clusters 2 and 3.

The transmission dynamics of Pestivirus is still not clearly explained by current knowledge of pestivirus infection, and uncertainties remain on possible management options. Three epidemiological patterns have been observed among monitored populations (Fig. 1)^45^, with outbreaks followed either by quick population recovery and decrease in virus circulation or by decreasing trends and an endemic situation of the infection, suggesting negative long-term impacts of the virus on population dynamics^45,46^. The virus has also been found to persist in some cases without any negative impact on population size^41^. Possible explanations for this variation include pathogen characteristics (*e.g*., variation in virulence), host populations (*e.g*., immunogenetic characteristics driving host susceptibility), as well as the environment (*e.g*., resource availability)^41^. In addition, whether this variation in transmission dynamics has consequences on the efficacy of management options remains unknown.

Our objective was to evaluate the role of demographic stochastic processes in governing epidemiological patterns of pestivirus spread in Pyrenean chamois populations, and to assess related implications for disease and population management. To address these issues, we developed a stochastic counterpart of a published deterministic model of pestivirus spread^43^. The modelling process benefits from a long-term demographic and epidemiological survey performed on the population of Pyrenean chamois in the Orlu natural reserve in France^40,47^. These empirical data offered the rare opportunity to combine capture-mark-recapture demographic estimates^48^ with serological surveys and advanced statistical modelling to give a realistic representation of the biological system. The deterministic version of the model already takes into account host seasonal ecology and behaviour, and thus adequately represents observed seasonal prevalence variation^43^ that may interact with management actions^49^. The stochastic version of the model has been developed to represent rare events and to predict virus fade-out, which has been observed in some populations^50^ and cannot be accounted for with a deterministic approach. First, we analysed predicted epidemiological patterns using cluster analysis of replications^51^. Second, using global sensitivity analysis^52^, we identified key parameters that influence virus persistence and epidemic size, in order to identify populational and environmental factors which, along with demographic stochasticity, contributed to explaining the variability of epidemiological dynamics. Third, we evaluated three management scenarios classically used in wildlife (*e.g*.^53,54^): modulation of non-selective culling (hunting), selective culling (test-and-cull of infected animals), and vaccination. In large herbivores, some species are the target of policies to conserve declining populations, while others are under exploitative management by hunting. To provide realistic management conclusions, and because these two contrasted cases exist in Pyrenean chamois, we evaluated disease management strategies in both hunted and protected populations.

## Results

### Model predictions and cluster analysis

**Figure 2:**
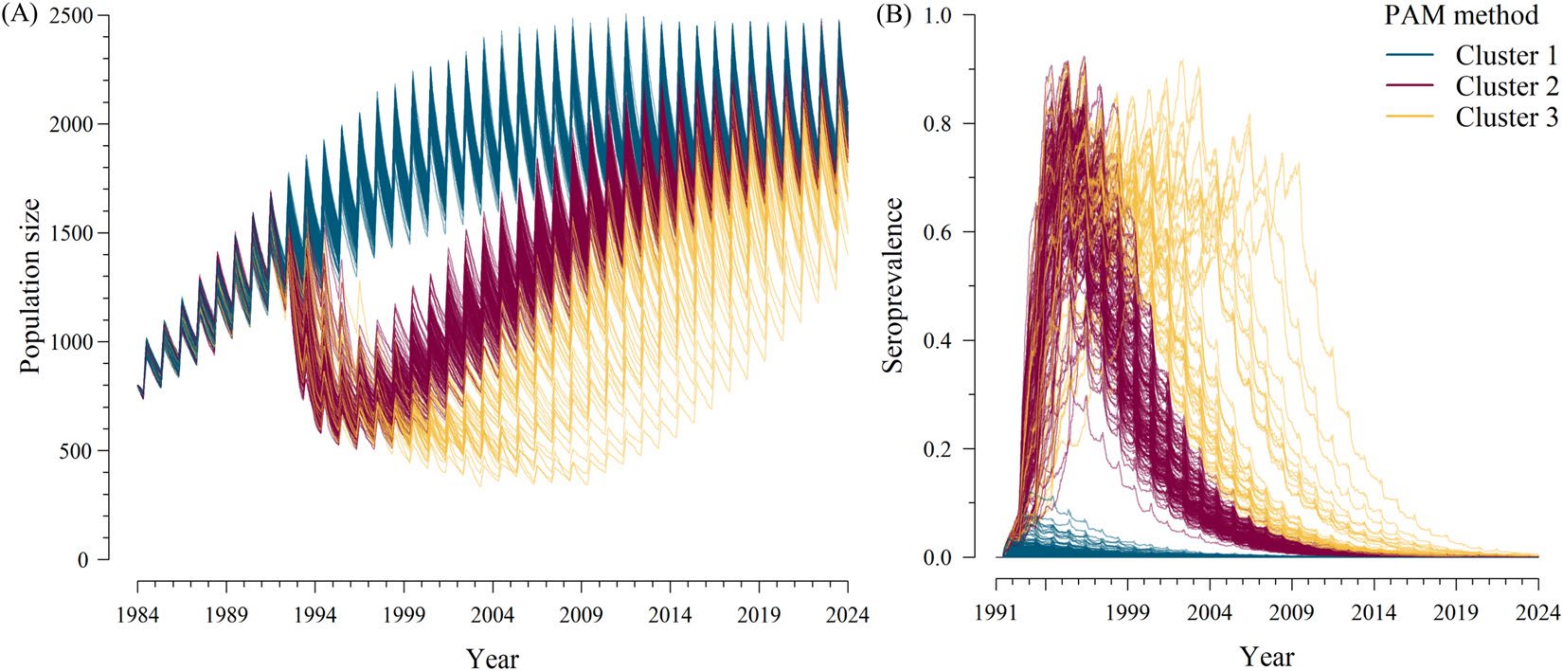
Evolution of population size (**A**) and seroprevalence (**B**) over simulation time for PAM clusters. Curves: model replications (400) of a scenario without management strategies and virus introduction in 1991, partitioned in three groups according to PAM algorithm (in blue: 220 replications in cluster 1, in purple: 140 replications in cluster 2, in yellow: 40 replications in cluster 3).

Figures 2A and 2B illustrate the results of 400 replications of the scenario without management strategies. Outcomes differed markedly after virus introduction in 1991. However, pestivirus infection faded out in all 400 replications less than 20 years after virus introduction. In half of the replications, extinction occurred ≤4 years after virus introduction.

**Figure 3:**
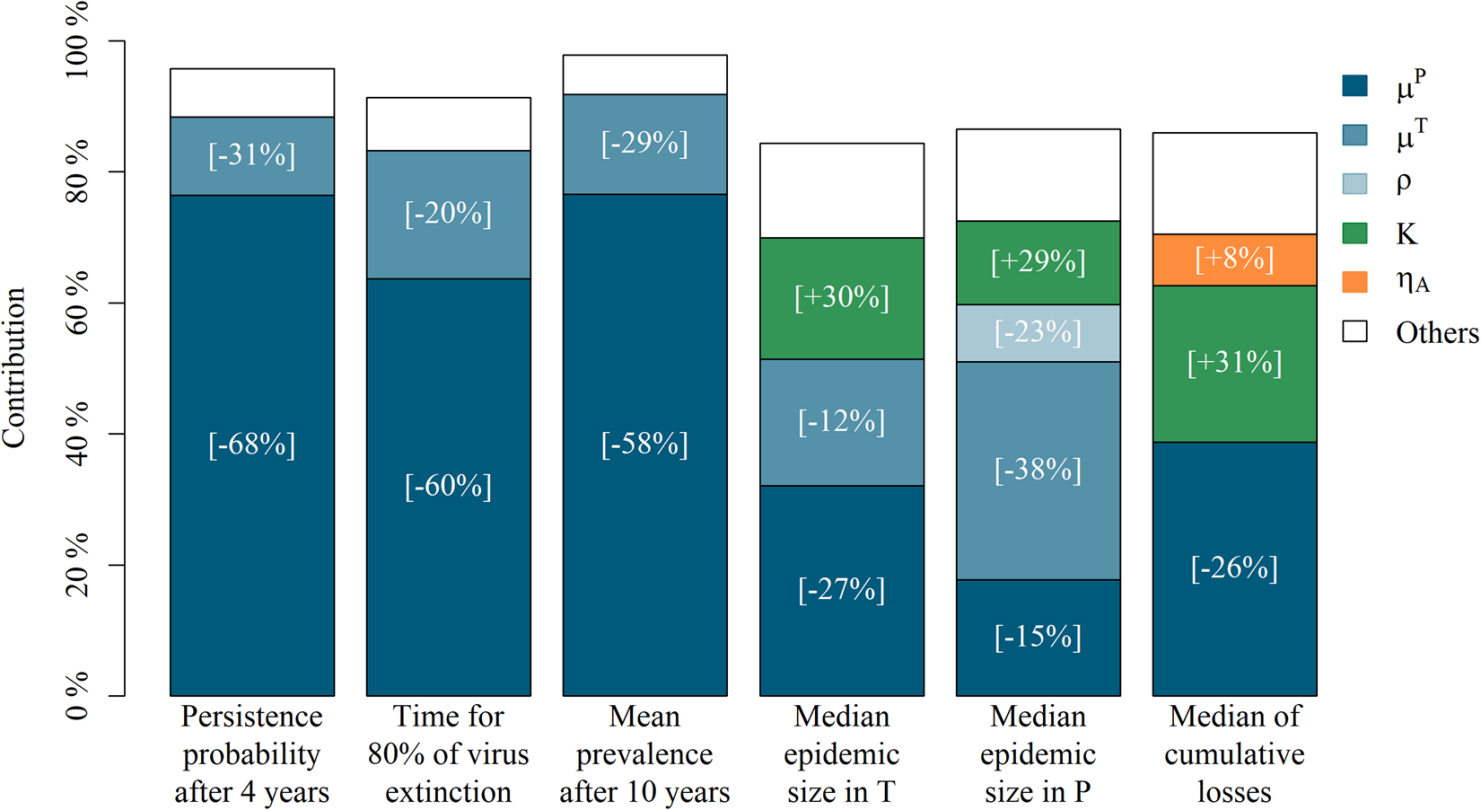
Contribution of model parameters to output variations and relative variation (in brackets) in each output induced by a 25% increase in each parameter. Parameters whose main effect or interaction with another parameter accounted for more than 5% of the output variance were retained. Parameters accounting for less than 5% of the output variance were grouped, and the sum of the contributions was equal to model *R*^2^. Six aggregated outputs were analysed: for all replications, including those in which the infection had faded-out, we considered the probability of virus persistence 4 years after virus introduction and the time after virus introduction needed to reach a probability of 80% of virus extinction in the population; for replications where the virus persisted more than 4 years after virus introduction, we considered the median cumulative epidemic size in *T* and *P* animals over the simulation time, the median cumulative number of infection-related losses over the simulation time, and the mean seroprevalence 10 years after virus introduction. Parameter definitions can be found in Table 2.

The optimal number of clusters using the average silhouette width was 3 for all methods, except for the single linkage method (see Supplementary Table S1 and Supplementary Figs S1 and 2). The discrimination of cluster 1 was similar with all methods, while distinction between clusters 2 and 3 was sensitive to the method but qualitatively similar (Table S1). We chose in the following to use PAM as the reference method for clustering (Table 1 and Fig. 2A and B), which was quantitatively the same as using complete linkage and UPGMA.

**Table 1:** Output values for cluster medoids with PAM and 80% credibility interval within each cluster (in brackets).

	Cumulative epidemic size in *P*	Cumulative number of infection-related losses	Time between virus introduction and its extinction (years)	Seroprevalence 10 years after virus introduction
Cluster 1 (220 replications)	0 [0–1]	24 [3–65]	0.9 [0.6–1.4]	0 [0–0.004]
Cluster 2 (140 replications)	35 [26–40]	1185 [982–1307]	6.2 [4.9–7.9]	0.18 [0.13–0.28]
Cluster 3 (40 replications)	46 [34–58]	1408 [1327–1857]	10.3 [8.9–14.8]	0.55 [0.39–0.77]

All methods distinguished a first cluster of 220 replications with a limited level of population invasion (maximal seroprevalence: 12%), few births of persistently infected (PI) animals over the duration of simulation (maximal cumulative number of PI animals: 4), an early fade-out of the virus (average duration of persistence less than one year), and no long-term impact on population growth (Table 1). The other two clusters corresponded to virus propagation associated with population decline: cluster 2 was associated with relatively short epidemics (average time to extinction: 6.2 years), while replications belonging to cluster 3 were characterized by longer persistence (average time to extinction: 10.3 years) and higher virus-related losses (Table 1 and Fig. 2A and B).

### Sensitivity analysis

Depending on the output considered, the parameters retained contributed between 84% and 98% to the output variance (Fig. 3). Aside from the interaction between the infection-related mortality of *T* (Transiently infected) animals *μ*^*T*^ and the infection-related mortality of *P* (Persistently infected) animals *μ*^*P*^ (see Table 2 for parameter definitions), no first order interactions accounted for more than 5% of the output variance. The main parameters contributing to output variation were *μ*^*P*^ (between 18% and 77% of output variance) and *μ*^*T*^ (between 12% and 33% of output variance). A 25% increase in these parameters induced marked relative decreases of the outputs (ranging from 12% to 68%).

The carrying capacity *K* was also found to partly contribute (between 13% and 24%) to the variance of three outputs: the median cumulative number of *T* and *P* animals, and the median cumulative number of infection-related losses (Fig. 3). A 25% increase of *K* induced an increase of about 30% of these outputs. Finally, the probability of abortion *ρ* and the fertility rate of adult females *η*_*A*_ had a less marked effect (<10%) on only 1 of the 6 outputs monitored.

### Management strategies

#### Efficacy of surveillance to detect epidemics

Efficacy of surveillance was measured as the number of replications in which management strategies were implemented divided by the number of replications in which there was a pestivirus epidemic (i.e. in which the virus did not fade-out early). In the protected population, efficacy was greater than 85% (range: 88–95%) when surveillance and/or carcass collection was high, and decreased to 27% when sampling effort was low for both protocols. In this context, low carcass collection (2.5% of the total population size) and high surveillance (20% of carcasses analyzed) appeared as the best compromise between sampling effort and efficacy. Management strategies were implemented in fewer replications in the case where populations were hunted, with a marked gain where more than half of the animals harvested were analyzed (efficacy of 60%) as compared to scenarios where only 15% were analyzed (efficacy of 11%).

#### Efficacy of management measures

**Figure 4:**
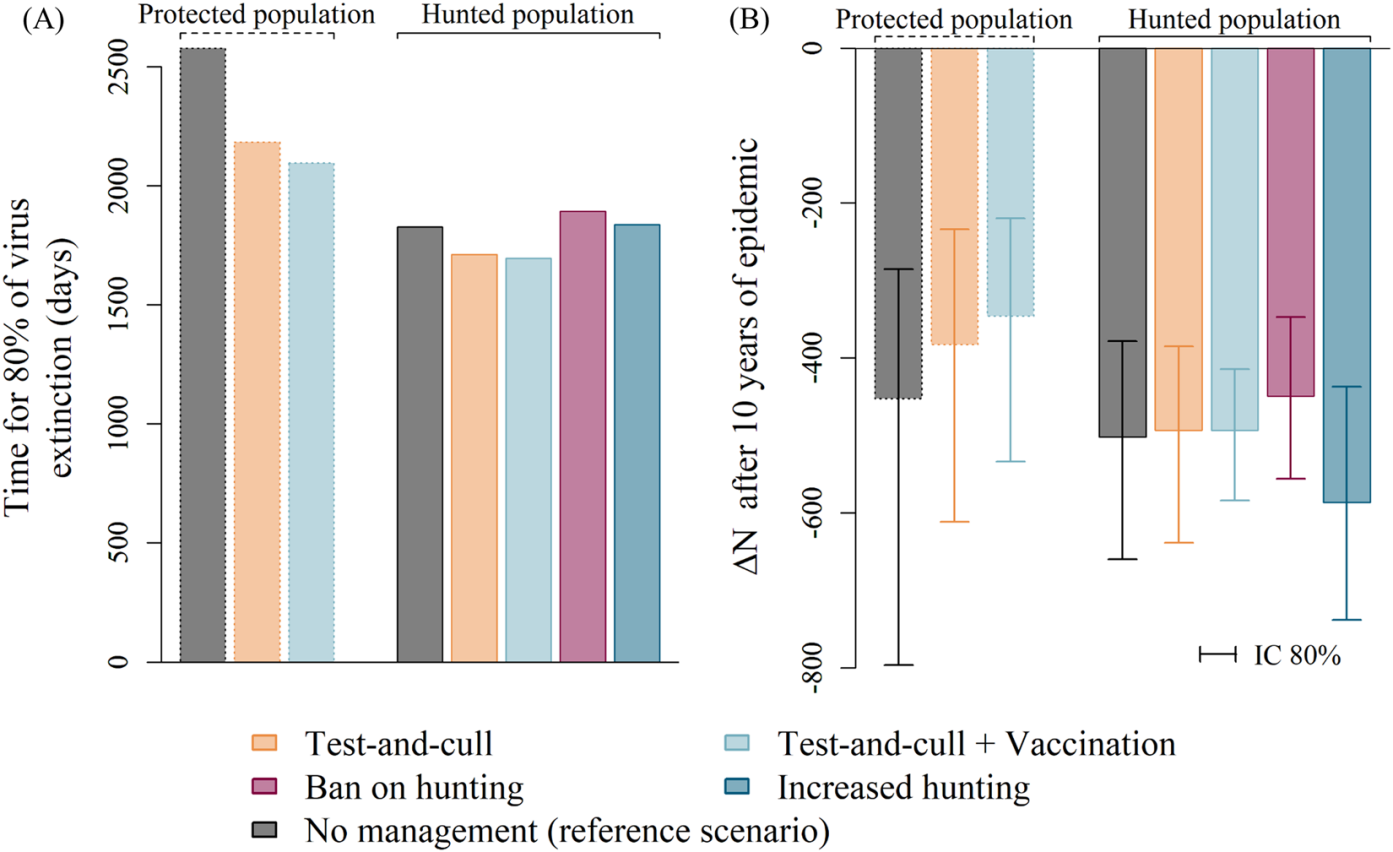
Outputs for management scenarios with test-and-cull and/or vaccination. Time after virus introduction needed to reach a probability of 80% of virus extinction in the population (**A**) and difference in population size (Δ*N*) after 10 years of epidemic for replications where the virus persisted more than 4 years after virus introduction (**B**), in two cases: protected population (dotted line) *vs* hunted population (solid line), for four scenarios: test-and-cull alone (orange), a combination of test-and-cull and vaccination (light blue), ban on hunting (purple) and increased hunting (darker blue). For each case (*i.e*., protected *vs* hunted), the reference scenario (*i.e*., no management method implemented) is represented in dark grey. Here, we used a capture rate of 30% and high surveillance and carcass collection (Table 3).

In the reference scenario (no management), the hunted and protected populations differed in terms of virus extinction time and epidemic size (Fig. 4): both outputs were lower in hunted than in protected populations. This is due to the difference in population size at virus introduction (larger in protected populations than in the hunted ones), given that both populations had the same initial conditions and a similar carrying capacity *K*.

Here, we chose to present the results for the highest sampling effort, *i.e*., a capture rate of 30% and high surveillance and carcass collection, so that effect sizes recorded for the different management scenarios are the strongest expected. In the protected population case, the duration of management measures (around 4 years) was similar for both strategies. Test-and-cull alone reduced the time needed to reach an 80% probability of virus extinction (−15.4%) and was only slightly more effective when combined with vaccination (−18.7%) (Fig. 4). Both methods enabled an increase in population growth (Δ*N*) over a 10-year period, helping speed-up population recovery (+15.4% and +23.5% respectively).

In the hunted population case, the duration of management measures was also around 4 years and was similar for all methods, except for a ban on hunting (3.7 years) and doubling the hunting rate (9.4 years), in relation with the criteria we used for stopping management measures.

Test-and-cull implemented alone or combined with vaccination contributed weakly and to the same extent to reducing the virus extinction time (−6.3% and −7.2% respectively, Fig. 4) and had virtually no effect (+1.8%) on the 10-year population size recovery.

The efficacy of modulation of hunting on virus extinction time was also low (<5%) and contrasted depending on the strategy: (−2.1% when targeting juveniles, +0.1% for subadults, +0.2% for adult males, +4.9% for adult females, +3.6% for ban on hunting and +0% for doubling the hunting rate). On the population size variation (Δ*N*) between 1991 and 2001, only a ban on hunting enabled an increase (+10.6%), whereas doubling the hunting rate induced a decrease (−16.7%), and targeted hunting had a much lower impact (−6.2% when targeting subadults, −3.6% for adult females, +2.1% for juveniles and +1% for adult males).

## Discussion

Stochastic variation is a crucial driver of the epidemiological dynamics in complex wildlife disease systems^14,55^. Here, the stochastic model distinguished three epidemiological dynamics: early fade-out where the virus did not invade the population and with no long-term population effect (cluster 1), epidemics with population decreases followed by virus extinction and population recovery (cluster 2), and epidemics with population decreases followed by endemic situations before the virus potentially faded out (cluster 3). These predictions were qualitatively distinct from the predictions issued from the deterministic model, that predicted an outbreak followed by an endemic situation^43^ and for which virus extinction did not belong to the possible outcomes. This difference is mainly due to demographic stochasticity, which can be explicitly accommodated in the new model. In particular, differences among clusters of simulated outcomes are related to variation in the number of PI births (10 to 48 in 4 years in clusters 2 and 3, versus less than 5 in cluster 1). This result, along with the importance of PI mortality in the sensitivity analysis, stresses the pivotal role of PI animals in pestivirus dynamics^43^. PI individuals are a specific feature of pestiviruses, and although they are uncommon, each of them may infect none to many conspecifics, so that their fate strongly influences epidemic dynamics. Interestingly, the situation predicted in cluster 1 (early fade-out) was predicted in more than half of the replications; thus short, unnoticed virus introductions may be frequent. However, we assumed here no further virus introduction. In real populations, repeated virus introductions may occur, which would decrease the probability of early fade-out, and thus the occurrence of cluster 1.

The dynamics predicted for clusters 2 and 3 closely matched those observed in the field in several Pyrenean chamois populations^45^. An interesting insight of the stochastic model was that it predicted virus extinction in all replications without management within 20 years of virus introduction (55% probability of extinction in less than 4 years), suggesting that pestivirus cannot persist over the long-term in the absence of virus reintroduction. However, this could also be a consequence of the assumption of spatial homogeneity, although we considered social segregation in the model (see 5.3). Spatial structure may be present either within the population if social groups are separated, or by considering surrounding populations in a metapopulation structure. Under a metapopulation structure, a long-term persistence is generally expected^56^. It would be interesting in the future to develop such a spatial model in order to explore the effect of population structure on virus persistence.

In contrast, it failed to represent the situation observed in one Spanish population (Freser-Setcases, Fig. 1), where seroprevalence is high (40% to 70%) but without any detrimental effects on population dynamics. This suggests that other drivers also contribute to virus persistence in Pyrenean chamois populations^41^. For example, population structure, low pathogen virulence^57^, genetic differences between host populations^58^, or high herd immunity before virus introduction (either due to cross-immunity with other pestivirus strains, such as border disease virus of ovine origin, or to residual immunity from a previous epidemic)^41^ could explain the situation observed in Freser-Setcases.

In addition to demographic stochasticity, populational and environmental factors that may further explain the variability of epidemiological dynamics were identified through the sensitivity analysis, by detecting key parameters affecting pestivirus persistence, epidemic size and population dynamics. Our analysis revealed that two demographic parameters, the mortality of transiently and persistently infected individuals and the carrying capacity have a major influence on epidemic sizes and their demographic consequences. Low mortality and high carrying capacity all predicted large epidemics with strong demographic impact especially when combined with high adult fertility. The rationale for this result is that these parameters contribute to the number of PI animals in the population, but also to the number of susceptible animals, which are mostly those between 0 and 2 years old^40^, through the impact of the carrying capacity *K* on density-dependent processes, especially the mortality of newborns *μ*_*B*_(*t*) and juveniles *μ*_*Juv*_(*t*). As a result, a high carrying capacity and a large population size at virus introduction, predicted larger epidemics, while a high carrying capacity and high fertility were associated with strong demographic consequences of epidemics. These predictions match the observed variation in the dynamics of classical swine fever in wild boar populations: local incidence and virus persistence were higher in sites where population size was large and/or close to habitat carrying capacity^59^. More generally, they illustrate the importance of host population dynamics as a key determinant in disease dynamics^7,60^.

Finally, we used our model to compare management strategies of pestivirus infection in Pyrenean chamois populations. Test-and-cull of infected animals is a major tool in managing pestivirus infections in domestic animals^61,62^. Here, its efficacy was low (<16%) with regard to virus extinction and for a trapping effort defined as the highest likely to be performed in natural populations. The combination of test-and-cull with vaccination was slightly better in protected populations, whereas the efficacy of both scenarios was similar in hunted populations. This contrast could be explained by (1) the fact that these scenarios were implemented for 0.6 years longer in protected than in hunted populations; (2) the fact that the reference level (virus extinction probability with no management action) was much lower in the hunted than in the protected population.

Models may be used to test scenarios that have not been implemented in the field, but that could be of more interest if they are predicted to be efficient. Here, vaccination was tested with this aim. Based on the demonstrated existence of cross-immunity between pestiviruses^63^, vaccines used against Bovine Viral Diarrhoea Virus in cattle are used in sheep. These vaccines induce a neutralizing antibody response in sheep^64^, but their efficacy against experimental infection has not yet been studied to our knowledge in small domestic ruminants, and *a fortiori* in wild-living small ruminants. We nevertheless assumed that an effective vaccine, providing long-lasting immunity, could be administered at high rates and during extended periods. Even considering these very optimistic assumptions in a context of wild populations, the efficacy of vaccination was low (<19%). This suggests that vaccination is not a relevant option to develop in the present epidemiological situation. A similar result has been found by Woodroffe^65^, who stressed that vaccination is most valuable in small populations with a very high risk of extinction, whereas in large populations, or over large areas, this treatment is often inappropriate or impractical, so that managing population size, structure or contact between host species could be a much better alternative. This conclusion could change if the epidemiological or population situation calls for strong efforts towards vaccination and if the proportion of effectively vaccinated animals can be elevated to high values^66^.

During a pestivirus epidemic, hunting tended to aggravate the situation, as demonstrated by the results of the scenario with a doubling of the hunting rate. Targeting hunting towards juveniles, while not particularly effective in reducing long-term virus persistence (<2%), was still the most effective type of selective harvest. This can be explained by the fact that targeting juveniles reduces the number of PI and susceptible animals. Targeting the other categories did not reduce virus persistence or improve population recovery. A ban on hunting, which is the most frequently used management option for Pyrenean chamois pestivirus^41^, reduced virus persistence only slightly. However, the population size increased between virus introduction and ten years afterwards, as recovery of population due to density-dependent processes (increase in subadult fertility and newborn and juvenile survival following decrease in population size due to virus-related mortality) was quicker if no hunting occurred. A ban on hunting when epidemics are detected thus appears to be the best compromise to improve population recovery. Again, this prediction confirms previous observations on the frequently counter-productive effect of hunting on the management of pestiviruses in wild ungulates^49,59^ and other diseases, in particular tuberculosis^67,68^. Multiple mechanisms have been involved in this effect of hunting^69^. Here, only density-dependent demographic and epidemiological processes have been accounted for, in particular the loss of herd immunity and weakening of density-dependence due to hunting-related mortality. Other effects, such as social and spatial reorganization, may also occur and worsen the influence of hunting^67^.

It is noteworthy that hunting had different effects when applied during periods of virus infection vs. when infection was not present. Generally speaking, non-hunted populations reach high densities and sustain large epidemics (*e.g*.^21^). Here, Pyrenean chamois populations that were hunted before virus entry, because of their relatively low population size compared to protected populations, were predicted to experience shorter epidemics. Thus, a generic management scenario for chamois populations would combine hunting in the absence of viruses, to limit population size, and a hunting ban during virus epidemics. When hunting is maintained, selectively targeting young animals appears as a potential solution to reduce virus persistence. Finally, hunting being the only way to gain information on virus transmission in hunted populations, it could be relevant to maintain surveillance by analysing hunter-harvested animals.

## Conclusions

Management options for pestivirus infection in Pyrenean chamois populations, as in many wild populations, are limited due to practical constraints, and our evaluation did not identify one absolute option that is both effective and feasible in practice. A reduction or ban on hunting could be the best and easiest method to reduce virus persistence and limit the decrease in population size in hunted populations. In protected areas where hunting cannot be adjusted, a ‘do nothing’ strategy could be recommended during epidemics considering the limited effects of treatments despite a surveillance effort known to be achieved only in the best-monitored populations ever, and as such probably far from what can be done from a routine perspective over large areas.

Finally, an important and unexpected aspect for the success of management was the importance of the surveillance scheme: management measures can be implemented only when surveillance allows managers to detect an epidemic. Here, the capacity of surveillance systems to detect epidemics varied according to the population considered. In protected populations such as in the Pyrenean National Park, a surveillance based on carcass collection combined with PCR analyses on found-dead animals was effective through either a combination of high carcass collection and a low surveillance protocol, or low carcass collection and high surveillance. Both protocols were almost as efficient as a combination of both high carcass collection and high surveillance. As a high carcass collection (such as in Gonzales and Crampe^32^) is difficult to achieve, maintaining a low level of carcass collection with a high number of analyses appears to be the most relevant option.

In hunted populations, which is the most frequent situation in the Pyrenees, the high surveillance protocol (60% of hunter-harvested animals analysed by PCR) was shown to be much more effective than the low one (15% of hunter-harvested animals analysed), and in any case less effective than the protocol based on found-dead animals used in protected populations. This suggests that surveillance based on hunter-harvested animals should maintain a high level of analyses and that hunting should not be stopped but only reduced as much as possible, in order to maintain the required level of surveillance. More generally, refining surveillance and monitoring schemes and analysing their cost and effectiveness may help to design relevant surveillance strategies that contribute both to management efficiency and knowledge improvement^70^.

## Material and methods

### Population monitoring

The data used in this study were collected from the population located in the National Game and Wildlife Reserve of Orlu in the eastern French Pyrenees (42.66°N, 1.97°E). The long-term Capture-Mark-Recapture (CMR) monitoring performed since 1984 made it possible to estimate age and sex-specific vital rates of the population^47^, and to provide individual data on pestivirus infection since 1995, when the epidemiological survey started^40^. Epidemiological data were also available on hunted animals during the same period (see^43^ for details). In addition, the minimum population size was annually estimated from a unique census performed in late June between 1984 and 2008. Although this approach systematically underestimates the true population size to a variable extent^71^, it should provide a relatively fair overview of the long-term trend in abundance of our population due to the very contrasted demographic periods encountered^43^. Monitoring of this population has been performed in accordance with ethical conditions of specific prefectural decree (n°2009-014) and in agreement with the French environmental code (Art. R421-15 to 31 and R422-92 to 94).

### Model design concepts and characteristics of the host-pathogen system

The model was structured in age classes and sexes and included three host characteristics of particular interest for assessing population demography: the seasonality of the reproduction cycle and contact structure of the Pyrenean chamois^72,73^; the density-dependence of newborn and juvenile survival and subadult fertility, which are known to be the first parameters to decrease at high density^74,75^; and the senescence processes affecting both survival and reproduction^47,76,77^. Introducing these characteristics in epidemiological models is essential as they can interact with management strategies, such as harvesting, and lead to compensatory mechanisms (*e.g*., increase in births) which may enhance transmission^8,49^.

The epidemiological structure of the model included the possibility of transient as well as persistent infections. Pestiviruses are characterized by congenital infection with fetuses infected in early gestation becoming Persistently Infected (PI) individuals, which excrete the virus lifelong and play a major role in spreading the disease. The existence of PI in Pyrenean chamois is suspected rather than proven, as this state is difficult to detect in the field. This is most likely due to the expected rarity of PI animals, their short lifespan and, above all, to the difficulty to differentiate between persistent and transient infection with animals found dead or captured, which can hardly be repeatedly monitored over time. However, PI birth from one experimentally infected female^78^ and virus detection in aborted foetuses^79^ led to suspect the possibility of vertical transmission in Pyrenean chamois. The role of PI animals in the disease spread was also demonstrated in a previous model, as model fit was much better when accounting for their presence^43^.

Transient infection induces severe clinical signs and long-lasting viraemia in Pyrenean chamois^80^. Accordingly, the associated mortality rate has been estimated as high^43^. Individuals that have recovered develop an immune response^80^, assumed to be long-lasting, as suggested by the observed increase in seroprevalence with age^40^. We assumed indirect transmission to be negligible because the virus has a limited survival time in the environment^61^.

### A stochastic epidemiological model in a structured and managed population

Starting from the deterministic model described in Beaunée *et al*.^43^, we performed three major model extensions. First, we accounted for demographic stochastic processes in order to model rare events and virus fade-out, which has been empirically reported in some populations^50^ and which is very likely to interact with management actions^49^. Second, we considered additional age classes and demographic estimates for old individuals in order to better account for senescence patterns and temporal variation in the age-structure of the population which determines the susceptibility of the population to demographic stochastic processes^81^. Third, we used this new model to provide the first insights on how different disease and population management strategies perform in both hunted and protected populations of Pyrenean chamois. The model was in discrete time with a time interval of 1 day. All simulations and further analyses were performed using R 3.2.2^82^.

The population was structured in six age classes: newborns *B* (May and June of the birth year), juveniles *J* (from 1^st^ of July to 30^th^ of June, *i.e*., [0–1[ year), subadults *Sa* ([1–2[ years), adults *A* ([2–8[ years), old adults *O* ([8–13[ years), and very old adults *VO* (≥13 years). As sexual maturity is reached from 18 to 20 months, a small proportion of females started breeding in *Sa*, while most started breeding in stage *A*^83,84^. Old and very old adults were modelled to account for senescence processes affecting both survival and reproduction^47,76,77^.

The density-dependence of the mortality of newborns and juveniles and the fertility of subadults were represented using sigmoid functions based on explicit variables (*d:* strength of density-dependence, *K*: carrying capacity, *N*: total population size; see Supplementary Equations S(28)–(30)).

**Figure 5:**
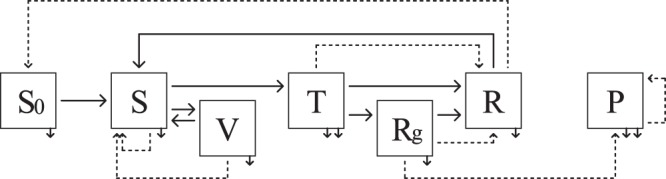
Simplified conceptual model of Pestivirus spread (adapted from Beaunée *et al*.^43^). Squares represent health states: *S*0, newborns and juveniles protected by maternal immunity, *S*, susceptible to infection, *T*, transiently infected, *R*, immune, *Rg*, immune females infected during pregnancy, *P*, persistently infected, and *V*, vaccinated. Solid horizontal arrows represent flows between health states (loss of maternal antibodies, infection, recovery, loss of acquired immunity, vaccination, and loss of vaccine-induced immunity). Solid vertical arrows represent natural mortality and disease-induced mortality for *T* and *P*. Dashed arrows represent births. See Supplementary Fig. S3 for the representation of the complete conceptual model.

We considered six health states (Fig. 5): *S*0, newborns and juveniles protected by maternal immunity, *S*, susceptible to infection, *T*, transiently infected (after horizontal transmission), *R*, immune, *P*, persistently infected (after vertical transmission), and *V*, vaccinated. A sub-state *Rg* was considered in state *R* to identify immune pregnant females infected during pregnancy. Females in *Rg* state remained *Rg* until the end of the birth period, then became *R*.

Newborn health state was determined according to the mother’s state at calving (Fig. 5 and Supplementary Fig. S3). *S* female gave birth to *S* calf, *T* female to *R* calf, and *R* female to *S*0 calf. Vertical transmission was assumed to occur during the first half of pregnancy and to lead to PI newborns or abortions^85^. Thus, an *Rg* female infected during the first half of pregnancy aborted with probability *ρ* (Table 2) or gave birth to a *P* calf. An *Rg* female infected during the second half of pregnancy gave birth to a *R* calf. A *P* female gave birth to a *P* calf, but this event was expected to be rare due to the low survival rate of PI individuals^43^. Vaccinated (*V*) female gave birth to *S* calf. To determine the number of PI births, the proportion of females in age class *X* (*Sa*, *A*, *O*, or *VO*) which became *Rg* at recovery and were transiently infected during the first half (*p*_*X*♀1_) or the second half (*p*_*X*♀2_) of pregnancy, was calculated at each time step (see Supplementary Equations S(20) and S(21)).

**Table 2:** Description and value of demographic and epidemiological parameters.

Description (dimension)		Value	References
**Demographic parameters**			
${\eta }_{Sa}^{max}$	Fertility rate of subadult females, maximum (births per female, annual)	0.30	*
*η*_*A*_	Fertility rate of adult females (births per female, annual)	0.90	^72^;*
*η*_*O*_	Fertility rate of old adult females (births per female, annual)	0.90	^72^;*
*η*_*VO*_	Fertility rate of very old adult females (births per female, annual)	0.50	*
${\mu }_{B}^{min}$	Probability of mortality of newborns, minimum (annual)	0.362	^77^
${\mu }_{B}^{max}$	Probability of mortality of newborns, maximum (annual)	0.85	^|^
${\mu }_{Juv}^{min}$	Probability of mortality of juveniles, minimum (annual)	0.218	*
${\mu }_{juv}^{max}$	Probability of mortality of juveniles, maximum (annual)	0.90	^|^
*μ*_*Sa*♀_	Probability of mortality of subadult females (annual)	0.052	*
*μ*_*Sa*♂_	Probability of mortality of subadult males (annual)	0.068	*
*μ*_*A*♀_	Probability of mortality of adult females (annual)	0.037	*
*μ*_*A*♂_	Probability of mortality of adult males (annual)	0.048	*
*μ*_*O*♀_	Probability of mortality of old adult females (annual)	0.078	*
*μ*_*O*♂_	Probability of mortality of old adult males (annual)	0.100	*
*μ*_*VO*♀_	Probability of mortality of very old adult females (annual)	0.146	*
*μ*_*VO*♂_	Probability of mortality of very old adult males (annual)	0.185	*
*δ*	Sex ratio	0.50	^†^
*K*	Carrying capacity	2,000	^†^
*d*	Strength of density dependence	0.0014	*
**Epidemiological parameters**			
1/*α*	Duration of maternal immunity (days)	60	^†^
1/*γ*	Duration of viraemia (days)	51	^79^
1/*ω*	Duration of acquired immunity (years)	8	^80^
*ρ*	Probability of abortion	0.50	^85^
*β*_*T*_	Horizontal transmission coefficient by a transiently infected animal (per day)	0.01	^‡^
*β*_*P*_	Horizontal transmission coefficient by a PI animal (per day)	0.48	^‡^
*μ* ^*T*^	Probability of mortality related to a transient infection (over the duration of viraemia)	0.76	^‡^
*μ* ^*P*^	Probability of mortality of PI animals (annual)	0.75	^90^

*Calibrated using field data.

^|^Calibrated to reach *K* at the disease-free equilibrium.

^†^Experts knowledge.

^‡^Approximate Bayesian Computation (see results in Supplementary Fig. S6).

The model accounted for the seasonality of the reproductive cycle^72^. During the mating season (from 5^th^ of November to 7^th^ of January), we assumed all individuals to be in contact (indicator of rut *τ* = 0). Outside this period (*τ* = 1), adult males formed a group separated from adult females, assuming no contact. Females stayed with juveniles and subadult females^73,86,87^. Subadult males were assumed to be in contact with both groups (see Supplementary Figs S4 and S5). The birth season (*ε* = 1) occurred from 30^th^ of April to 1^st^ of July^88^ (*ε* = 0 otherwise). Hence, the gestation period (*ν* = 1) lasted from 5^th^ of November (beginning of the mating season) to 1^st^ of July (end of the birth season).

We modelled three management strategies: (1) modulation of non-selective culling (hunting), including ban of hunting, (2) selective culling (test-and-cull), and (3) vaccination. Test-and-cull and vaccination were assumed to be performed during monitoring by CMR. Hunting season lasted from 1^st^ of September to 30^th^ of November (indicator of hunt *ϕ* = 1). Capture season lasted from 1^st^ of April to 30^th^ of November (indicator of capture *θ* = 1). Parameter values associated with management strategies (probability of mortality related to hunting *μ*^*H*^, probability of mortality related to test-and-cull for *T* and *P* animals *μ*^*HTP*^, and vaccination of susceptible animals *v*) are described thereafter (§5.8 Management strategies and Table 3).

**Table 3:** Description and value of management-related parameters (*N*: total population size, *J*: number of juveniles, *Sa*: number of subadults, *A*_♀_: number of adult females (≥2 years), *A*_♂_: number of adult males (≥2 years)).

Description (dimension)		Value
**Capture parameters**		
*μ* ^*HTP*^	Capture rate	[0.02; 0.3]
${\mu }_{Juv}^{HTP}$	Probability of test & cull-related mortality of juveniles (over the duration of capture period)	*μ*^*HTP*^ * 0.07 * *N*/*J*
${\mu }_{Sa}^{HTP}$	Probability of test & cull-related mortality of subadults (over the duration of capture period)	*μ*^*HTP*^ * 0.17 * *N*/*Sa*
${\mu }_{A\male}^{HTP}$	Probability of test & cull-related mortality of adult females (≥ 2years) (over the duration of capture period)	${\mu }^{HTP}\,\ast \,0.59\,\ast \,N/{A}_{\male}$
${\mu }_{A\female}^{HTP}$	Probability of test & cull-related mortality of adult males (≥ 2years) (over the duration of capture period)	${\mu }^{HTP}\,\ast \,0.16\,\ast \,N/{A}_{\female}$
*v*_*Juv*_	Probability of vaccination of juveniles (over the duration of capture period)	*μ*^*HTP*^ * 0.07 * *N*/*J*
*v*_*Sa*_	Probability of vaccination of subadults (over the duration of capture period)	*μ*^*HTP*^ * 0.17 * *N*/*Sa*
*ν*_*A*♀_	Probability of vaccination of adult females (≥ 2years) (over the duration of capture period)	${\mu }^{HTP}\,\ast \,0.59\,\ast \,N/{A}_{\male}$
*ν*_*A*♂_	Probability of vaccination of adult males (≥ 2years) (over the duration of capture period)	${\mu }^{HTP}\,\ast \,0.16\,\ast \,N/{A}_{\female}$
1/*λ*	Duration of vaccine-induced immunity (years)	2
**Hunting parameters**		
*μ* ^*H*^	Harvest rate	0.1
${\mu }_{Juv}^{H}$	Probability of hunting-related mortality of juveniles (over the duration of hunting period)	*μ*^*H*^ * 2/6 * *N*/*J*
${\mu }_{Sa}^{H}$	Probability of hunting-related mortality of subadults (over the duration of hunting period)	*μ*^*H*^ * 1/6 * *N*/*Sa*
${\mu }_{A\male}^{H}$	Probability of hunting-related mortality of adult females (≥ 2years) (over the duration of hunting period)	${\mu }^{H}\,\ast \,1/6\,\ast \,N/{A}_{\male}$
${\mu }_{A\female}^{H}$	Probability of hunting-related mortality of adult males (≥ 2years) (over the duration of hunting period)	${\mu }^{H}\,\ast \,2/6\,\ast \,N/{A}_{\female}$
**Boolean parameters**		
*τ*	Indicator of rut	0/1
*ε*	Indicator of birth	0/1
*ν*	Indicator of gestation	0/1
*θ*	Indicator of capture	0/1
*ϕ*	indicator of hunt	0/1

### Calibration of uncertain parameters

Parameter values (Table 2) were calibrated in Beaunée *et al*.^43^. However, some parameter values were modified to account for new analyses performed using data collected from the long-term CMR monitoring of the Orlu population (Unpublished data). To obtain age-specific estimates of the probability of natural mortality, we fitted a global capture-mark-resighting model on all CMR data collected from 1984 to 2016^48^. All males and females aged between 0 and 20 years at first capture (n = 388 and 208 respectively) were included in the model. Survival probabilities were modelled as an additive function of sex, age classes (*J*, *Sa*, *A*, *O* and *VO*) and period (before or after 1991, when virus introduction has been estimated by^43^) and resighting probabilities as an additive function of sex and year. Model estimates were obtained using RMark 2.2.2^89^. Age-specific survival estimates obtained during the period before virus introduction (<1991; Table 2) were those used in our stochastic model.

The carrying capacity *K* was calibrated using experts’ knowledge and set at 2,000 animals. Maximum probabilities of newborn and juvenile mortality and the strength of density dependence *d* were calibrated so that the disease-free equilibrium of the population was *K* and to match the time series of counts performed in Orlu before virus introduction.

Epidemiological parameters with the least information for their calibration were the rate of horizontal transmission by transiently infected animals, horizontal transmission by PI animals, and probability of transient infection-related mortality. These three parameters were estimated using a deterministic counterpart of the updated model, applying Approximate Bayesian Computation (ABC) approach and data as described in Beaunée *et al*.^43^ (see results in Supplementary Fig. S6). The probability of mortality of PI animals and the probability of abortion remain unknown in Pyrenean chamois and thus were based on available knowledge in domestic ruminants^85,90^. Similarly, very few information is available regarding the duration of maternal immunity that we calibrated based on experts’ knowledge (Table 2). Finally, the duration of viremia and the duration of acquired immunity were based on empirical and experimental knowledge on Pyrenean chamois^79,80^.

### Stochastic equations for model transitions

Transitions between compartments were modelled as stochastic flows assuming demographic stochasticity. Possible transitions were: mortality Μ, loss of maternal antibodies Δ, infection Υ, recovery Γ, loss of acquired immunity Ω, vaccination Θ, loss of vaccine-induced immunity Λ, hunting Φ, and test-and-cull Φ^*TP*^. Each was the outcome of a binomial trial. For multiple transitions from a given compartment, multinomial distributions were used.

Each flow (1, …, *j*) from compartment *i* was associated with daily rate *κ*_*ij*_(*t*). The probability associated with each event *j* was^91^: ${p}_{ij}=[1-exp(-\sum _{j\ne i}{\kappa }_{ij})]\cdot {\kappa }_{ij}/\sum _{j\ne i}{\kappa }_{ij}$, with ${p}_{ii}=1-\,\sum _{j\ne i}{p}_{ij}$ the probability of staying in compartment *i*.

Births Η followed a binomial distribution with probability 1 − *exp*(−*η*). As newborn orphans are expected to have a very low survival rate, only breeding females still alive at birth time were considered.

The complete system of mathematical equations is given in Supplementary Equations S(1)–(30). The transitions between age groups were not included in these equations because they were considered as deterministic discrete events happening each 1^st^ of July, with every *B*, *J*, and *Sa* individual going into the next age class (*J*, *Sa*, and *A* respectively), 1/6 of *A* becoming *O* (the adult stage lasting 7 − 2 + 1 = 6 years), and 1/5 of *O* becoming *VO* (the old adult stage lasting 12 − 8 + 1 = 5 years).

### Initial conditions and model outputs

The model ran for 40 years starting in 1984, when yearly censuses started. The initial population composition (age and sex classes) was based on the stable structure observed at disease-free equilibrium with the updated deterministic counterpart of the model without management strategies. The initial population size was 800 individuals (minimum population size in 1984 estimated from ground counts), and all animals were susceptible. The virus introduction corresponded to the birth of a PI individual in the middle of the birth period in 1991^43^. The virus was assumed not to be further reintroduced.

We evaluated disease spread, pathogen persistence and its effect on population through the following model outputs: epidemic size (cumulative number of *T* or *P* animals over the simulation time), cumulative number of infection-related losses (infection-related deaths of *T* and *P* animals and abortions), difference in population size (Δ*N*) between virus introduction and 10 years afterwards, time (in days) between virus introduction and fade-out (assumed to occur when there was no longer a *T* or *P* animal, and no longer an *Rg* female), and seroprevalence 10 years after virus introduction. In addition, we evaluated management-related outputs (§5.8 Management strategies): duration of management measures and importance of sampling effort (number of hunter-harvested animals or number of carcasses found, and number of analyses performed).

To facilitate analyses, we considered the following aggregate outputs:

- For all replications, including those with early infection fade-out, we considered the probability of virus persistence 4 years after virus introduction (*i.e*., number of replications in which the virus persisted among the total number of replications) and the time needed after virus introduction to reach a probability of 80% of virus extinction in the population.
- For replications where the virus persisted over more than 4 years, we considered the median cumulative epidemic size in *T* and *P* animals and the median cumulative number of infection-related losses over the simulation time, as well as the mean seroprevalence 10 years after virus introduction.

### Cluster and sensitivity analyses

To assess if demographic stochasticity drives epidemiological patterns, we performed a cluster analysis of model stochastic repetitions. We used 400 repetitions of a scenario without management strategies, and calculated the distance between each pair of repetitions using normalized Euclidean distance for the following outputs: cumulative number of *P* animals, cumulative number of infection-related losses, number of days between virus introduction and fade-out, and seroprevalence 10 years after virus introduction.

We compared five hierarchical methods commonly used and available in function *hclust* from STATS R package^82^: single linkage, complete linkage, Unweighted Pair-Group Method using arithmetic Averages or UPGMA, Unweighted Pair-Group Method using Centroids or UPGMC, and Ward’s method, as well as one non-hierarchical method available in CLUSTER R package^92^, Partitioning Around Medoids or PAM. The use of average silhouette width^93^ allowed us to optimize the number of clusters and to compare results from the different methods^94^. Silhouette width measures the degree of membership of an object to its cluster and varies from −1 to 1. It can be averaged over all objects of a partition; a high average silhouette indicates strong support for the partition.

We were also interested in identifying parameters that most influenced epidemiological dynamics. To do so, we performed a global sensitivity analysis using a fractional factorial design^95^. Input parameters were 15 demographical parameters (carrying capacity *K*, probability of mortality *μ* for the 10 age/sex classes, and fertility rate *η* for the 4 age classes) and 8 epidemiological parameters (duration of maternal immunity 1/*α*, duration of viraemia 1/*γ*, duration of acquired immunity 1/*ω*, probability of abortion *ρ*, both horizontal transmission coefficients *β*_*X*_, and probabilities of mortality related to transient infection *μ*^*T*^ and to permanent infection *μ*^*P*^)_._ We used 3 levels per parameter: 75%, 100%, and 125% of their nominal value (except for rates *η*_*A*_ and *η*_*O*_ which were bounded at 1 because chamois are uniparous). Using PLANOR R package^96^, we generated a fractional factorial plan of resolution V, which allowed us to estimate all of the main effects and first order interactions assuming that higher order interactions are negligible^97,98^. This design represents 3^8^ = 6561 scenarios. 400 replications were performed per scenario. All aggregate outputs were considered.

For each output, a linear regression model was run with all main effects and first order interactions. Parameters whose total contribution accounted for more than 5% of the output variance were retained. This total contribution of factor *i* to variation in output *y* was calculated as ${C}_{i}^{y}=(S{S}_{i}^{y}+\frac{1}{2}\sum _{j\ne i}S{S}_{i:j}^{y})\,{\slash}S{S}_{tot}^{y}$ where $S{S}_{i}^{y}$ is the sum of squares related to the main effect of factor *i*, $S{S}_{i:j}^{y}$ the sum of squares related to the first-order interaction between factors *i* and *j*, and $S{S}_{tot}^{y}$ the total sum of squares^52^. The sum of the contributions equalled model *R*^2^.

### Management strategies

We contrasted two cases when evaluating management strategies: a protected population in which hunting was forbidden, and a hunted population in which harvest rate was 10% of the population size. In doing so, we expected our results to be relevant for a wide range of ungulate species, including Pyrenean chamois, for which these two management situations are commonly encountered.

In both cases, management measures were implemented the year after population managers identified a population decline associated with virus detection. The criteria for identifying a pestivirus-related population decline was the detection of two infected (viraemic) animals during the same year associated with a 30% decrease in population size over the last two years. Hence, measures could not be implemented in replications where the virus faded out early with no long-term impact on population size. Measures were stopped when no infected animal was detected for 1 year and population size was stable for two consecutive years.

In the protected population case, surveillance was modelled assuming PCR virus detection on carcasses of found-dead animals and management strategies were based on capture-recapture protocol. Carcass collection was performed between November and April, with either high carcass collection rate as observed in the Pyrenean National Park (10% of the estimated population size^32^), or low carcass collection rate (2.5%). PCR was realized on a proportion of these carcasses, with either a high surveillance rate (20% of the carcasses analysed) or a low surveillance rate (5%). We used these proportions because it is much more difficult to obtain good-quality samples (blood or spleen) for PCR analysis in found-dead carcasses than in hunter-harvested carcasses. Two distinct strategies were implemented during captures: either test-and-cull of infected animals, or a combination of test-and-cull of infected animals with vaccination of susceptible ones. From data collected in the Orlu population, we considered the proportion of the population captured each year to vary between 2% and 30% and to be distributed as follow: 7% juveniles, 17% subadults, 59% adult females, and 16% adult males.

In the hunted population case, the surveillance protocol was modelled as PCR virus detection on carcasses of harvested animals, and management strategies included modulation of the hunting effort. PCR was realized on a proportion of hunted animals, with either high (60% of carcasses analysed) or low (15%) surveillance rate. Five different strategies were implemented: (i) test-and-cull during captures, (ii) test-and-cull combined with vaccination during captures, (iii) targeted hunting (specific age and sex class), (iv) ban on hunting and (v) increased hunting. The proportion of population captured (for test-and-cull and vaccination) followed the same distribution as in the protected population case. Harvest rate was 10% of the population size, and the distribution of harvested animals mimics the one commonly found in French Pyrenees: 33% juveniles, 17% subadults, 17% adult females, and 33% adult males. Adult females and adult males were pooled irrespective of their age, because it is not possible to easily differentiate among adults, old adults, and very old adults in the field. Targeted hunting strategy induced a modification of this distribution, with 50% of the harvested animals being of the target class (4 possible targets: juveniles, subadults, adult females, adult males), and 17% for each of the three other classes. We also tested a scenario of hunting intensification, considering a doubling of the hunting rate.

## Electronic supplementary material

Supplementary Information: Tables, Figures and Equations

## Data Availability

The datasets analysed during the current study are available in^43^ and within its Supplementary Information files.
