# Supplementary material for: Demographic stochasticity drives epidemiological patterns in wildlife with implications for diseases and population management
Source: Sci Rep. 2018 Nov 15;8:16846. doi: 10.1038/s41598-018-34623-0 (PMC6237989; doi:10.1038/s41598-018-34623-0)
Supplement: Supplementary file 1 — Supplementary Information: Tables, Figures and Equations [file 41598_2018_34623_MOESM1_ESM.pdf]

Demographic stochasticity drives epidemiological patterns in wildlife with implications for diseases and population management

Sébastien Lambert, Pauline Ezanno, Mathieu Garel and Emmanuelle Gilot-Fromont

Supplementary Information: Tables, Figures and Equations

Table S1: Number of clusters and average silhouette width for different clustering methods

		Hierarchical					*Non-hierarchical*
		Single linkage[1]	Complete linkage[2]	UPGMA[3]	UPGMC[3]	Ward[4]	PAM[5]
**Optimal number of clusters**		2	3	3	3	3	3
**Average silhouette width**		0.79	0.81	0.81	0.81	0.81	0.81
**Number of replications**	Cluster 1	220	220	220	220	220	220
	Cluster 2	180	140	140	143	140	140
	Cluster 3	-	40	40	37	31	40

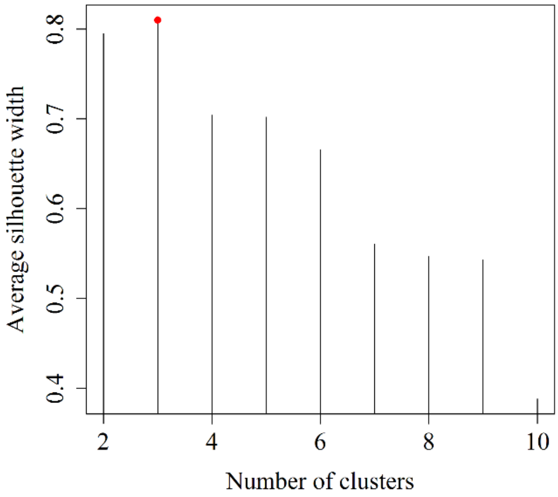

Figure S1: Average silhouette width plotted against number of clusters. The highest average silhouette width corresponding to the optimum number of clusters is represented with a red dot.

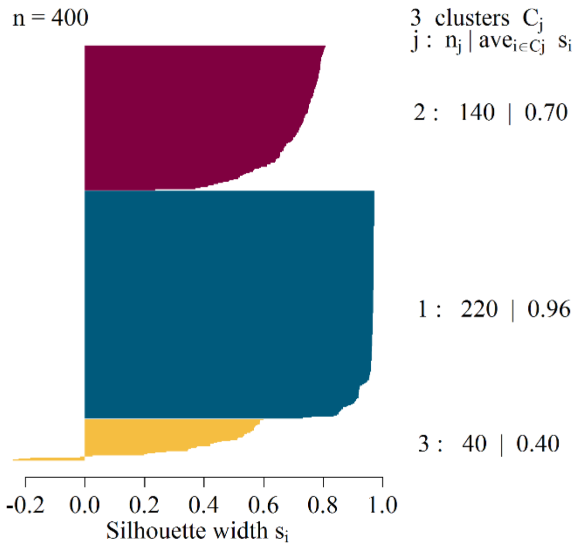

Figure S2: Silhouette plot obtained with 3 clusters using PAM. For each cluster j, the number of replications and the average silhouette width of the cluster are given on the right.

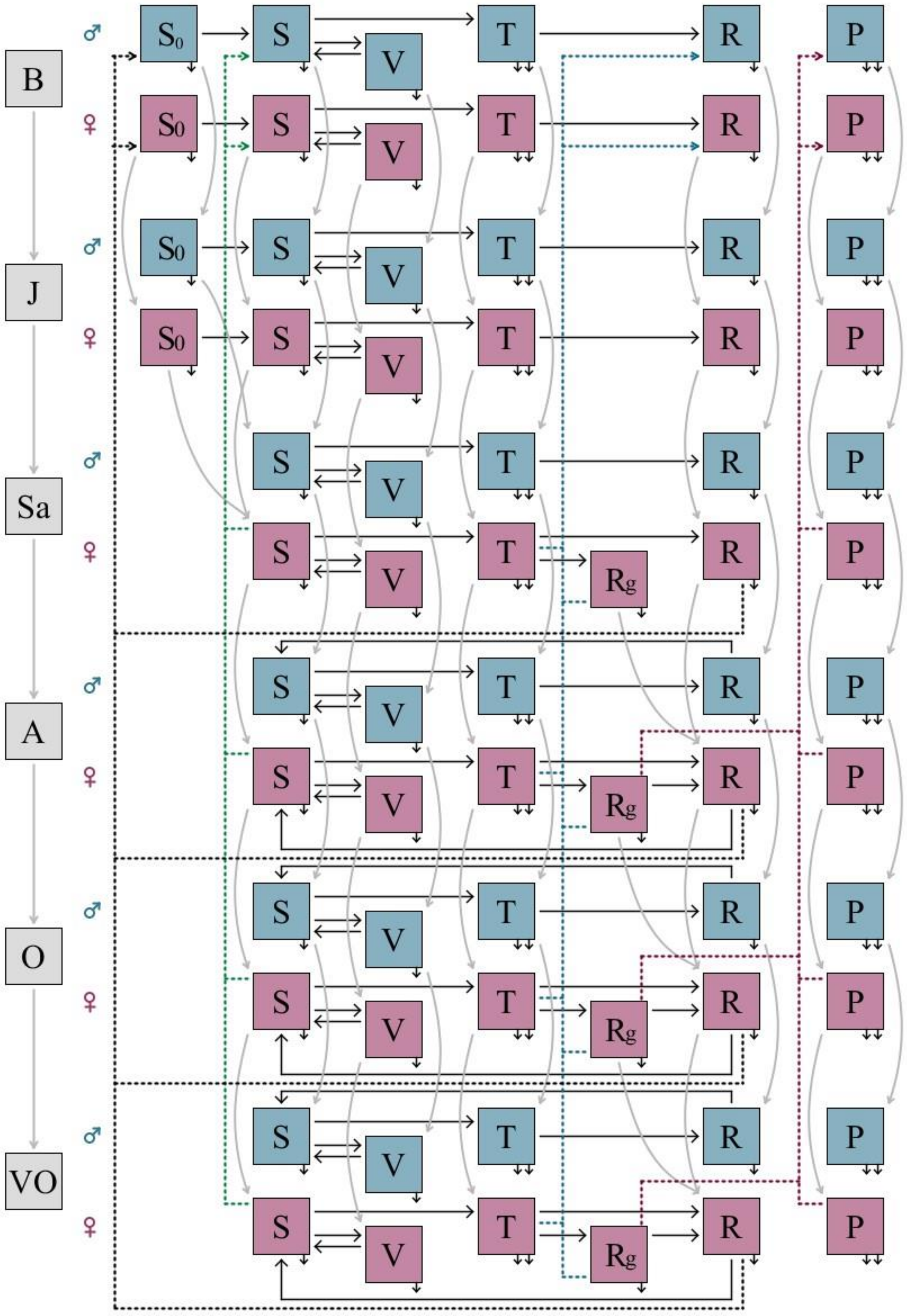

Figure S3: Complete conceptual model of Pestivirus spread (adapted from Beaunée *et al.*, 2015[6]). Squares represent health states: $S0$, newborns and juveniles protected by maternal immunity, S, susceptible to infection, T, transiently infected, R, immune, Rg, immune females infected during pregnancy, P, persistently infected and V, vaccinated. Solid horizontal arrows represent flows between compartments (loss of maternal antibodies, infection, recovery, loss of acquired immunity, vaccination, and loss of vaccine-induced immunity). Solid vertical arrows represent natural mortality and disease-induced mortality for T and P. Solid grey arrows represent transitions between age classes. Dashed arrows represent births.

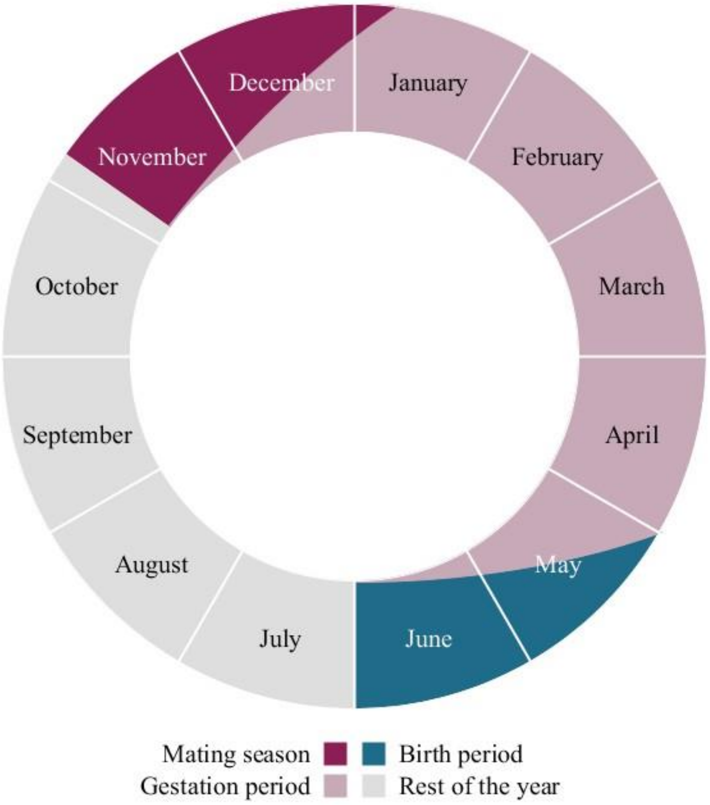

Figure S4: Graphical representation of seasonality. Mating season started the 5th of November and ended the 7th of January ($\tau = 0$), birth period started the 30th of April and ended the 1st of July ($\varepsilon = 1$), and consequently the gestation period ($\nu = 1$) lasted from the 5th of November (beginning of mating season) to the 1st of July (end of birth season).

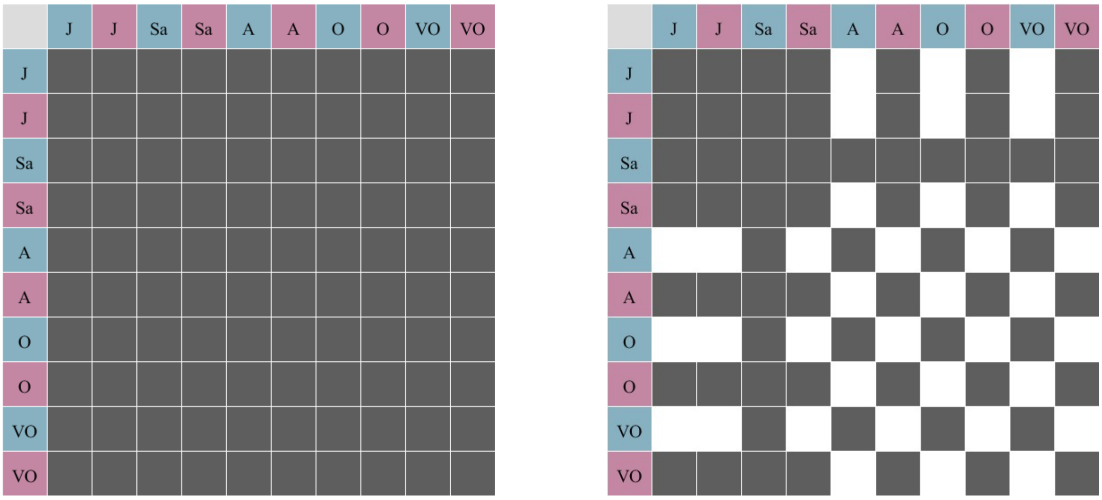

Figure S5: Matrix contacts based on sex, age and season (adapted from Beaunée *et al.*, 2015[6]). During mating season on the left, and out of mating season on the right. In black: possible contact, in white: no contact. Age classes: juveniles *J*, subadults *Sa*, adults *A*, old adults *O*, very old adults *VO*. Sex: males in blue and females in red. During mating season, $\tau = 0$ because all individuals were in contact (on the left), and out of mating season $\tau = 1$ because adult males (*A*, *O* and *VO*) form a separate group, adult females stay with juveniles and subadults females, and subadult males were in contact with both groups.

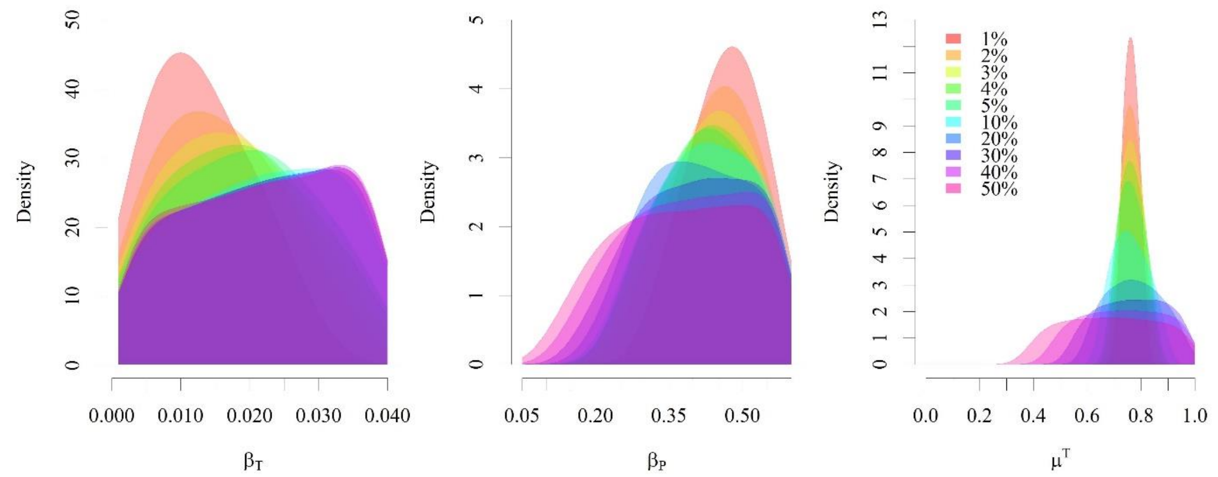

Figure S6: Distribution of probability density of parameter values for 1%, 2%, 3%, 5%, 10%, 20%, 30%, 40% and 50% of simulations producing lowest chi-square distance between observed and simulated data using Approximate Bayesian Computation (ABC) approach and data as described in Beaunée *et al.* (2015)[6]. Parameter values corresponded to the 1% threshold (median and 95% confidence intervals): the coefficient of horizontal transmission from transiently infected animals $\beta_T = 0.01[0.002; 0.028]$, the coefficient of horizontal transmission from persistently infected animals $\beta_P = 0.48[0.33; 0.59]$ and the disease-related mortality of transiently infected animals $\mu^T = 0.76[0.72; 0.81]$.

Equations S(1)-(30): Model equations

Transitions between compartments were modelled as stochastic flows assuming demographic stochasticity. Possible transitions were: mortality M, loss of maternal antibodies Δ, infection ϒ, recovery Γ, loss of acquired immunity Ω, vaccination Θ, loss of vaccine-induced immunity Λ, hunting Φ and test-and-cull Φ^{TP}. Each was the outcome of a binomial trial. For multiple transitions from a given compartment, multinomial distributions were used.

Each flow $(1,\ldots,j)$ from a compartment i was associated with a daily rate $\kappa_{ij}(t)$. The probability associated with each event j was $p_{ij} = \left[1 - exp\left(-\sum_{j\neq i}\kappa_{ij}\right)\right].\kappa_{ij}/\sum_{j\neq i}\kappa_{ij}$ (Bretó *et al.*, 2009[7]), with $p_{ii} = 1 - \sum_{j\neq i} p_{ij}$ the probability of staying in compartment i.

Births H followed a binomial distribution with probability $1 - exp(-\eta)$. As newborn orphans are expected to have a very low survival rate, only breeding females still alive at birth time were considered.

The complete system of mathematical equations describing the updating of variables corresponding to health states at each time step is given below (1), with X the class age, and ⚥ the symbol used when equations are the same between males ♂ and females ♀.

The transitions between age groups were not included in these equations because they were considered as deterministic discrete events happening each 1st of July, with every B, J, and Sa individuals going into the next age class (J, Sa and A respectively), $1/6$ of A becoming O (the adult age lasting $7 - 2 + 1 = 6$ years) and $1/5$ of O becoming VO (the old adult age lasting $12 - 8 + 1 = 5$ years).

$$
\begin{aligned}
&S0_{X⚥}(t+1) = S0_{X⚥}(t) - \mathrm{M}_{S0X⚥}(t) - \Phi_{S0X⚥}(t) - \Delta_{X⚥}(t) + \mathrm{H}_{S0X⚥}(t)\\
&S_{X⚥}(t+1) = S_{X⚥}(t) - \mathrm{M}_{SX⚥}(t) - \Phi_{SX⚥}(t) - \Upsilon_{X⚥}(t) - \Theta_{X⚥}(t) + \Delta_{X⚥}(t) + \Lambda_{X⚥}(t)\\
&\qquad\qquad + \Omega_{X⚥}(t) + \mathrm{H}_{SX⚥}(t)\\
&V_{X⚥}(t+1) = V_{X⚥}(t) - \mathrm{M}_{VX⚥}(t) - \Phi_{VX⚥}(t) - \Lambda_{X⚥}(t) + \Theta_{X⚥}(t)\\
(1)\quad &T_{X⚥}(t+1) = T_{X⚥}(t) - \mathrm{M}_{TX⚥}(t) - \mathrm{M}^{T}_{TX⚥}(t) - \Phi_{TX⚥}(t) - \Phi^{TP}_{TX⚥}(t) - \Gamma_{X⚥}(t) + \Upsilon_{X⚥}(t)\\
&R_{X♂}(t+1) = R_{X♂}(t) - \mathrm{M}_{RX♂}(t) - \Phi_{RX♂}(t) - \Omega_{X♂}(t) + \Gamma_{X♂}(t) + \mathrm{H}_{RX♂}(t)\\
&R_{X♀}(t+1) = R_{X♀}(t) - \mathrm{M}_{RX♀}(t) - \Phi_{RX♀}(t) - \Omega_{X♀}(t) + \left(1 - \nu(t)\right).\Gamma_{X♀}(t) + \mathrm{H}_{RX♀}(t)\\
&Rg_{X♀}(t+1) = Rg_{X♀}(t) - \mathrm{M}_{RgX♀}(t) - \Phi_{RgX♀}(t) + \nu(t).\Gamma_{X♀}(t)\\
&P_{X⚥}(t+1) = P_{X⚥}(t) - \mathrm{M}_{PX⚥}(t) - \mathrm{M}^{P}_{PX⚥}(t) - \Phi_{PX⚥}(t) - \Phi^{TP}_{PX⚥}(t) + \mathrm{H}_{PX⚥}(t)
\end{aligned}
$$

With: $\exists(V,\Phi,\Theta,\Lambda) \Leftrightarrow X \neq B$; $\exists(S0,\Delta) \Leftrightarrow X \in \{B,J\}$;
$\exists\mathrm{H} \Leftrightarrow X = B$
$\exists R_g \Leftrightarrow X \in \{Sa,A,O,VO\}$; $\exists\Omega \Leftrightarrow X \in \{A,O,VO\}$

The equations describing flows are given below for each health state (2-19). Parameter descriptions are given in Table 2 of the article.

$\boldsymbol{S0}$**:** Individuals protected by maternal antibodies were affected by three events: natural mortality M_{S0}, hunting Φ_{S0} (except for newborns) and loss of maternal antibodies Δ. We used the notation $\overline{S0}$ for the event in which individuals stayed in compartment $S0$, with $\overline{S0} = \overline{(\mathrm{M}_{S0} \cup \Phi_{S0} \cup \Delta)}$.

(2) *Newborns:* $(\mathrm{M}_{S0B},\ \Delta_B, \overline{S0}) \sim Multinomial\left(S0_B,\ p_1,\ p_2, 1-\sum_{j=1}^{2} p_j\right)$

With: $\forall j \in [1,2],\ p_j = \kappa_j . \frac{1-exp\left[\sum_{j=1}^{2}\kappa_j\right]}{\sum_{j=1}^{2}\kappa_j}$ And: $\kappa = [\mu_B(t), \alpha]$

(3) *Juveniles:* $\left(\mathrm{M}_{S0J}, \Phi_{S0J},\ \Delta_J, \overline{S0}\right) \sim Multinomial\left(S0_J,\ p_1,\ p_2,\ p_3, 1-\sum_{j=1}^{3} p_j\right)$

With: $\forall j \in [1,3],\ p_j = \kappa_j . \frac{1-exp\left[\sum_{j=1}^{3}\kappa_j\right]}{\sum_{j=1}^{3}\kappa_j}$ And: $\kappa = \left[\mu_{Juv}(t), \phi(t).\mu_{Juv}^{H}, \alpha\right]$

***S*:** Susceptible individuals were affected by the following events; natural mortality M_S, hunting Φ_S, transient infection Υ and vaccination Θ. We used the notation $\bar{S}$ for the event in which individuals stayed in compartment S, with $\bar{S} = \overline{(\mathrm{M}_S \cup \Phi_S \cup \Upsilon \cup \Theta)}$.

The horizontal transmission function depended on sex and age of individuals and took into account changes in contact patterns due to the mating season through the Boolean parameter τ defined in §2.2 and Table 1 of the article. Thus, according to the contact matrix (see Supplementary Fig. S6), three forces of infection were considered (4-6): f_1 for juveniles (females and males) and breeding females (subadult to very old adult age classes), f_2 for subadult males and f_3 for adult males. N is the total population, every other capital letter refers to total number of either a class age (independent of the health state) or a health state (from the whole population or in a specific class of age and sex as indicated in subscripts). The transmission function was assumed to be frequency-dependent.

(4) $$f_1 = \left(\frac{\beta_T.\left(T-\tau(t).T_{(A+O+VO)♂}\right) + \beta_P.\left(P-\tau(t).P_{(A+O+VO)♂}\right)}{N-\tau(t).\left(A_{♂}+O_{♂}+VO_{♂}\right)}\right)$$

(5) $$f_2 = \left(\beta_T.\frac{T}{N} + \beta_P.\frac{P}{N}\right)$$

(6) $$f_3 = \left(\frac{\beta_T.\left[T-\tau(t).\left(T_{B+J+(Sa+A+O+VO)♀}\right)\right] + \beta_P.\left[P-\tau(t).\left(P_{B+J+(Sa+A+O+VO)♀}\right)\right]}{N-\tau(t).\left(B+J+Sa_{♀}+A_{♀}+O_{♀}+VO_{♀}\right)}\right)$$

(7) *Newborns:* $(\mathrm{M}_{SB},\ \Upsilon_B, \bar{S}) \sim Multinomial\left(S_B,\ p_1,\ p_2, 1-\sum_{j=1}^{2} p_j\right)$

With: $\forall j \in [1,2],\ p_j = \kappa_j . \frac{1-exp\left[\sum_{j=1}^{2}\kappa_j\right]}{\sum_{j=1}^{2}\kappa_j}$ And: $\kappa = [\mu_B(t), f_1]$

(8) *Juveniles and breeding females* ($X = \{J, Sa_{♀}, A_{♀}, O_{♀}, VO_{♀}\}$)*:*

$(\mathrm{M}_{SX},\ \Phi_{SX},\ \Upsilon_X,\ \Theta_X, \bar{S}) \sim Multinomial\left(S_X,\ p_1,\ p_2,\ p_3,\ p_4, 1-\sum_{j=1}^{4} p_j\right)$

With: $\forall j \in [1,2],\ p_j = \kappa_j . \frac{1-exp\left[\sum_{j=1}^{2}\kappa_j\right]}{\sum_{j=1}^{2}\kappa_j}$ And: $\kappa = [\mu_B(t), f_1]$

(9) *Subadult males:*

$(\mathrm{M}_{SSa♂}, \Phi_{SSa♂}, \Upsilon_{Sa♂}, \Theta_{Sa♂}, \bar{S}) \sim Multinomial\left(S_{Sa♂},\ p_1,\ p_2,\ p_3,\ p_4, 1-\sum_{j=1}^{4} p_j\right)$

With: $\forall j \in [1,4],\ p_j = \kappa_j . \frac{1-exp\left[\sum_{j=1}^{4}\kappa_j\right]}{\sum_{j=1}^{4}\kappa_j}$ And: $\kappa = [\mu_{Sa♂}, \phi(t).\mu_{Sa}^{H}, f_2, \theta(t).v_{Sa♂}]$

(10) *Adult males* ($X = \{A_{♂}, O_{♂}, VO_{♂}\}$)*:*

$(\mathrm{M}_{SX}, \Phi_{SX}, \Upsilon_X, \Theta_X, \bar{S}) \sim Multinomial\left(S_X,\ p_1,\ p_2,\ p_3,\ p_4, 1-\sum_{j=1}^{4} p_j\right)$

With: $\forall j \in [1,4],\ p_j = \kappa_j . \frac{1-exp\left[\sum_{j=1}^{4}\kappa_j\right]}{\sum_{j=1}^{4}\kappa_j}$ And: $\kappa = [\mu_X, \phi(t).\mu_{A♂}^{H}, f_3, \theta(t).v_{A♂}]$

$\boldsymbol{V}$: Vaccinated individuals were affected by natural mortality M_V, hunting Φ_V and loss of vaccine-induced immunity Λ. We used the notation $\bar{V}$ for the event in which individuals stayed in compartment V, with $\bar{V} = \overline{(\mathrm{M}_V \cup \Phi_V \cup \Lambda)}$.

(11) *Juveniles, Subadults and Adults* ($X = \{J, Sa_{♀}, Sa_{♂}, A_{♀}, A_{♂}, O_{♀}, O_{♂}, VO_{♀}, VO_{♂}\}$):

$$(\mathrm{M}_{VX}, \Phi_{VX}, \Lambda_{VX}, \bar{V}) \sim Multinomial\left(V_X, p_1, p_2, p_3, 1 - \textstyle\sum_{j=1}^{3} p_j\right)$$

$$\text{With: } \forall j \in [1,3],\ p_j = \kappa_j . \frac{1 - exp\left[\sum_{j=1}^{3} \kappa_j\right]}{\sum_{j=1}^{3} \kappa_j} \quad \text{And:} \kappa = [\mu_X, \phi(t).\mu_X^H, \lambda]$$

$\boldsymbol{T}$: Transiently infected animals were affected by natural mortality M_T, infection-related mortality M_T^T, hunting Φ_T, test-and-cull Φ_T^{TP} and recovery Γ. We used the notation $\bar{T}$ for the event in which individuals stayed in compartment T, with $\bar{T} = \overline{(\mathrm{M}_T \cup \mathrm{M}_T^T \cup \Phi_T \cup \Phi_T^{TP} \cup \Gamma)}$.

(12) *Newborns:* $(\mathrm{M}_{TB}, \mathrm{M}_{TB}^T, \Gamma_B, \bar{T}) \sim Multinomial\left(T_B, p_1, p_2, p_3, 1 - \sum_{j=1}^{3} p_j\right)$

$$\text{With: } \forall j \in [1,3],\ p_j = \kappa_j . \frac{1 - exp\left[\sum_{j=1}^{3} \kappa_j\right]}{\sum_{j=1}^{3} \kappa_j} \quad \text{And:} \kappa = [\mu_B(t), \mu^T, \gamma]$$

(13) *Juveniles, Subadults and Adults* ($X = \{J, Sa_{♀}, Sa_{♂}, A_{♀}, A_{♂}, O_{♀}, O_{♂}, VO_{♀}, VO_{♂}\}$) :

$$(\mathrm{M}_{TX}, \mathrm{M}_{TX}^T, \Phi_{TX}, \Phi_{TX}^{TP}, \Gamma_X, \bar{T}) \sim Multinomial\left(T_X, p_1, p_2, p_3, p_4, p_5, 1 - \textstyle\sum_{j=1}^{5} p_j\right)$$

$$\text{With: } \forall j \in [1,5],\ p_j = \kappa_j . \frac{1 - exp\left[\sum_{j=1}^{5} \kappa_j\right]}{\sum_{j=1}^{5} \kappa_j} \quad \text{And:} \kappa = [\mu_X, \mu^T, \phi(t).\mu_X^H, \theta(t).\mu_X^{HTP}, \gamma]$$

$\boldsymbol{R}$: Recovered individuals (immune) were affected by natural mortality M_R, hunting Φ_R and loss of acquired immunity Ω (adults only). We used the notation $\bar{R}$ for the event in which individuals stayed in compartment R, with $\bar{R} = \overline{(\mathrm{M}_R \cup \Phi_R \cup \Omega)}$.

(14) *Newborns:* $\mathrm{M}_{RB}(t) \sim Bin(R_B(t), 1 - exp[-\mu_B(t)])$

(15) *Juveniles and Subadults* ($X = \{J, Sa_{♀}, Sa_{♂}\}$):

$$(\mathrm{M}_{RX}, \Phi_{RX}, \bar{R}) \sim Multinomial\left(R_X, p_1, p_2, 1 - \textstyle\sum_{j=1}^{2} p_j\right)$$

$$\text{With: } \forall j \in [1,2],\ p_j = \kappa_j . \frac{1 - exp\left[\sum_{j=1}^{2} \kappa_j\right]}{\sum_{j=1}^{2} \kappa_j} \quad \text{And:} \kappa = [\mu_X, \phi(t).\mu_X^H]$$

(16) *Adults* ($X = \{A_{♀}, A_{♂}, O_{♀}, O_{♂}, VO_{♀}, VO_{♂}\}$) :

$$(\mathrm{M}_{RX}, \Phi_{RX}, \Omega_X, \bar{R}) \sim Multinomial\left(R_X, p_1, p_2, p_3, 1 - \textstyle\sum_{j=1}^{3} p_j\right)$$

$$\text{With: } \forall j \in [1,3],\ p_j = \kappa_j . \frac{1 - exp\left[\sum_{j=1}^{3} \kappa_j\right]}{\sum_{j=1}^{3} \kappa_j} \quad \text{And:} \kappa = [\mu_X, \phi(t).\mu_X^H, \omega]$$

$\boldsymbol{Rg}$: Immune females infected during pregnancy ($X = \{Sa_{♀}, A_{♀}, O_{♀}, VO_{♀}\}$) were affected by natural mortality M_{Rg} and hunting Φ_{Rg}. We used the notation $\overline{Rg}$ for the event in which individuals stayed in compartment Rg, with $\overline{Rg} = \overline{(\mathrm{M}_{Rg} \cup \Phi_{Rg})}$.

(17) $(\mathrm{M}_{RgX}, \Phi_{RgX}, \overline{Rg}) \sim Multinomial\left(Rg_X, p_1, p_2, 1 - \sum_{j=1}^{2} p_j\right)$

$$\text{With: } \forall j \in [1,2],\ p_j = \kappa_j . \frac{1 - exp\left[\sum_{j=1}^{2} \kappa_j\right]}{\sum_{j=1}^{2} \kappa_j} \quad \text{And:} \kappa = [\mu_X, \phi(t).\mu_X^H]$$

***P*:** Permanently infected animals were affected by natural mortality M_P and infection-related mortality M_P^P, hunting Φ_P and test and cull Φ_P^{TP}. We used the notation $\bar{P}$ for the event in which individuals stayed in compartment P, with $\bar{P} = \overline{(M_P \cup M_P^P \cup \Phi_P \cup \Phi_P^{TP})}$.

(18) *Newborns:* $(M_{PB}, M_{PB}^P, \bar{P}) \sim Multinomial\left(P_B, p_1, p_2, 1 - \sum_{j=1}^{2} p_j\right)$

With: $\forall j \in [1,2],\ p_j = \kappa_j . \dfrac{1 - exp\left[\sum_{j=1}^{2} \kappa_j\right]}{\sum_{j=1}^{2} \kappa_j}$ And: $\kappa = [\mu_B, \mu^P]$

(19) *Juveniles, Subadults and Adults* ($X = \{J, Sa_{♀}, Sa_{♂}, A_{♀}, A_{♂}, O_{♀}, O_{♂}, VO_{♀}, VO_{♂}\}$)*:*

$(M_{PX}, M_{PX}^P, \Phi_{PX}, \Phi_P^{TP}, \bar{P}) \sim Multinomial\left(P_X, p_1, p_2, p_3, p_4, 1 - \sum_{j=1}^{2} p_j\right)$

With: $\forall j \in [1,4],\ p_j = \kappa_j . \dfrac{1 - exp\left[\sum_{j=1}^{4} \kappa_j\right]}{\sum_{j=1}^{4} \kappa_j}$ And: $\kappa = [\mu_X, \mu^P, \phi(t).\mu_X^H, \theta(t).\mu_X^{HTP}]$

Equations describing births for each health state are given below, with $X = \{Sa, A, O, VO\}$ (22-27). The number of females infected during the first half (mothers of P newborns) and second half (mothers of R newborns) of pregnancy are (20-21):

(20) *1st half:* $\psi_{RgX♀1}(t) \sim Bin((p_{X♀1} + p_{X♀2}).\left[Rg_{X♀}(t) - M_{RgX♀}(t) - \Phi_{RgX♀}(t)\right], p_{X♀1})$

(21) *2nd half:* $\psi_{RgX♀2}(t) = (p_{X♀1} + p_{X♀2}).\left[Rg_{X♀}(t) - M_{RgX♀}(t) - \Phi_{RgX♀}(t)\right] - \psi_{RgX♀1}(t)$

$p_{X♀1}(t)$ was the proportion of females in class X (Sa, A, O, or VO) that became Rg after recovery and were transiently infected during the first half of pregnancy (16). Pregnancy lasted 170 days (Serrano *et al.*, 2015[8]). Females who became infected up to 51 days before pregnancy were included because the virus was still present when they got pregnant.

$p_{X♀2}(t)$ was the proportion of females in class X (Sa, A, O, or VO) that became Rg after recovery and were transiently infected during the second half of gestation (17). Females that became infected less than 51 days before giving birth were not included because they became R and not Rg (immunity was acquired after birth).

$T_{N,X♀}(t)$ was the number of new cases of transiently infected individuals at time t (incidence) in females of class X (Sa, A, O, or VO).

(22) $$p_{X♀1}(t) = \frac{\sum_{k=t-(170+51)}^{k=t-86} T_{N,X♀}(k)}{\sum_{k=t-(170+51)}^{k=t-51} T_{N,X♀}(k)}$$ (23) $$p_{X♀2}(t) = \frac{\sum_{k=t-85}^{k=t-51} T_{N,X♀}(k)}{\sum_{k=t-(170+51)}^{k=t-51} T_{N,X♀}(k)}$$

As long as no female became infected during pregnancy (denominator null), $p_{X♀1}$ and $p_{X♀2}$ were null and $\psi_{RgX♀1} = \psi_{RgX♀2} = 0$. In the other cases, $p_{X♀1}(t) + p_{X♀2}(t) = 1$ so $\psi_{RgX♀1} + \psi_{RgX♀2} = Rg_{X♀}(t) - M_{RgX♀}(t) - \Phi_{RgX♀}(t)$.

(24) $\boldsymbol{SO}$

$H_{RX \to SO}(t) \sim Bin(R_{X♀}(t) - M_{RX♀}(t) - \Phi_{RX♀}(t), 1 - exp[-\varepsilon(t).\eta_X])$

Females ♀: $H_{SOB♀}(t) \sim Bin(H_{RSa \to SO}(t) + H_{RA \to SO}(t) + H_{RO \to SO}(t) + H_{RVO \to SO}(t), \delta)$

Males ♂: $H_{SOB♂}(t) = H_{RSa \to SO}(t) + H_{RA \to SO}(t) + H_{RO \to SO}(t) + H_{RVO \to SO}(t) - H_{SOB♀}(t)$

(25) ***S***

$\mathrm{H}_{SX\to S}(t) \sim Bin(S_{X♀}(t) - \mathrm{M}_{SX♀}(t) - \Phi_{SX♀}(t), 1 - exp[-\varepsilon(t).\eta_X])$

$\mathrm{H}_{VX\to S}(t) \sim Bin(V_{X♀}(t) - \mathrm{M}_{VX♀}(t) - \Phi_{VX♀}(t), 1 - exp[-\varepsilon(t).\eta_X])$

Females ♀: $\mathrm{H}_{SB♀}(t) \sim Bin\left(\sum_{X\in\{Sa,A,O,VO\}} \mathrm{H}_{SX\to S}(t) + \sum_{X\in\{Sa,A,O,VO\}} \mathrm{H}_{VX\to S}(t), \delta\right)$

Males ♂: $\mathrm{H}_{SB♂}(t) = \sum_{X\in\{Sa,A,O,VO\}} \mathrm{H}_{SX\to S}(t) + \sum_{X\in\{Sa,A,O,VO\}} \mathrm{H}_{VX\to S}(t) - \mathrm{H}_{SB♀}(t)$

(26) ***R***

$\mathrm{H}_{TX\to R}(t) \sim Bin(T_{X♀}(t) - \mathrm{M}_{TX♀}(t) - \mathrm{M}^{T}_{TX♀}(t) - \Phi_{TX♀}(t) - \Phi^{TP}_{TX♀}(t), 1 - exp[-\varepsilon(t).\eta_X])$

$\mathrm{H}_{RgX\to R}(t) \sim Bin(\psi_{RgX♀2}, 1 - exp[-\varepsilon(t).\eta_X])$

Females ♀: $\mathrm{H}_{RB♀}(t) \sim Bin\left(\sum_{X\in\{Sa,A,O,VO\}} \mathrm{H}_{TX\to R}(t) + \sum_{X\in\{Sa,A,O,VO\}} \mathrm{H}_{RgX\to R}(t), \delta\right)$

Males ♂: $\mathrm{H}_{RB♂}(t) = \sum_{X\in\{Sa,A,O,VO\}} \mathrm{H}_{TX\to R}(t) + \sum_{X\in\{Sa,A,O,VO\}} \mathrm{H}_{RgX\to R}(t) - \mathrm{H}_{RB♀}(t)$

(27) ***P***

$\Psi_{PX}(t) \sim Bin(P_{X♀}(t) - \mathrm{M}_{PX♀}(t) - \mathrm{M}^{P}_{PX♀}(t) - \Phi_{PX♀}(t) - \Phi^{TP}_{PX♀}(t), 1 - exp[-\varepsilon(t).\eta_X])$

$\mathrm{H}_{PX\to P}(t) \sim Bin(\Psi_{PX}(t), 1 - \rho)$

$\Psi_{RgX}(t) \sim Bin(\psi_{RgX♀1}, 1 - exp[-\varepsilon(t).\eta_X])$

$\mathrm{H}_{RgX\to P}(t) \sim Bin\left(\Psi_{RgX}(t), 1 - \rho\right)$

Females ♀: $\mathrm{H}_{PB♀}(t) \sim Bin\left(\sum_{X\in\{Sa,A,O,VO\}} \mathrm{H}_{PX\to P}(t) + \sum_{X\in\{Sa,A,O,VO\}} \mathrm{H}_{RgX\to P}(t), \delta\right)$

Males ♂: $\mathrm{H}_{PB♂}(t) = \sum_{X\in\{Sa,A,O,VO\}} \mathrm{H}_{PX\to P}(t) + \sum_{X\in\{Sa,A,O,VO\}} \mathrm{H}_{RgX\to P}(t) - \mathrm{H}_{PB♀}(t)$

Equations describing density-dependent parameters are given below (28-30):

Probability of newborn and juvenile mortality as well as the subadult fertility rate were affected by density-dependence. For these parameters, we used a sigmoid function using explicit variables (d: strength of density-dependence, K: carrying capacity, N: total population size). In the case of the subadult fertility rate, we considered the population size at the year of birth, *i.e.*, two years before.

(28) $\mu_B(t) = \mu_B^{min} + \dfrac{\mu_B^{max} - \mu_B^{min}}{1 + exp(K * d - d * N(t))}$

(29) $\mu_{Juv}(t) = \mu_{Juv}^{min} + \dfrac{\mu_{Juv}^{max} - \mu_{Juv}^{min}}{1 + exp(K * d - d * N(t))}$

(30) $\eta_{Sa}(t) = \dfrac{\eta_{Sa}^{max}}{1 + exp(d * N(t - 2\, years) - d * K)}$

References:

1. Sneath, P. H. The application of computers to taxonomy. *J. Gen. Microbiol.* **17,** 201–226 (1957).
2. Sorensen, T. A method of establishing groups of equal amplitude in plant sociology based on similarity of species content and its application to analyses of the vegetation on Danish commons. *Biol. Skr.* **5,** 1–34 (1948).
3. Sneath, P. H. A. & Sokal, R. R. *Numerical taxonomy. The principles and practice of numerical classification.* (Freeman, 1973).
4. Ward, J. H. Hierarchical grouping to optimize an objective function. *J. Am. Stat. Assoc.* **58,** 236–244 (1963).
5. Kaufman, L. & Rousseeuw, P. J. *Finding groups in data: an introduction to cluster analysis*. (Wiley, 2005).
6. Beaunée, G., Gilot-Fromont, E., Garel, M. & Ezanno, P. A novel epidemiological model to better understand and predict the observed seasonal spread of Pestivirus in Pyrenean chamois populations. *Vet. Res.* **46,** 86 (2015).
7. Bretó, C., He, D., Ionides, E. L. & King, A. A. Time series analysis via mechanistic models. *Ann. Appl. Stat.* **3,** 319–348 (2009).
8. Serrano, E. *et al.* Border Disease Virus: an exceptional driver of chamois populations among other threats. *Virology* **6,** 1–9 (2015).
